# Aging strong: an N = 1 mixed-methods study of long-term supervised high-intensity resistance training in a 71-year-old woman

**DOI:** 10.3389/fragi.2026.1844227

**Published:** 2026-06-01

**Authors:** Itai Har-Nir, Noam Manor, Jana Pelclova

**Affiliations:** 1 Institute of Active Lifestyle, Faculty of Physical culture, Palacký University, Olomouc, Olomouc, Czech Republic; 2 Department of Physiotherapy, Faculty of Health Sciences, Ariel University, Shomron, Israel

**Keywords:** healthy aging, high-intensity resistance training, load-velocity profiling, mixed-methods, muscle strength, older adults

## Abstract

Older adults who engage in long-term, supervised high-intensity resistance training (HIRT) may preserve musculoskeletal function, independence, and psychological resources beyond typical age-related expectations. This N = 1 mixed-methods study characterized an upper-bound example of “aging strong” in a 71-year-old woman after eight consecutive years of structured HIRT. The participant completed three 60-min HIRT sessions per week emphasizing multi-joint lifts performed close to muscular failure, alongside high daily ambulatory activity and a protein-rich diet. A convergent assessment battery integrated dual-energy X-ray absorptiometry (DXA), one-repetition maximum tests, load–velocity and load–power profiling, functional performance tests (Short Physical Performance Battery, Timed Up and Go, 30-s chair stand, push-ups to failure), validated questionnaires of general self-efficacy and self-esteem, and a semi-structured interview analyzed using reflexive thematic analysis. DXA showed low whole-body fat (15.1%) and a high appendicular lean mass index (10.33 kg·m^‐2^), exceeding age-matched reference values and sarcopenia thresholds. Maximal strength was exceptional (bench- and leg-press one-repetition maximum of 1.05× and 3.85× body weight, respectively), and functional performance indicated minimal disability risk (Short Physical Performance Battery 12/12, Timed Up and Go 6.08 s, 30-s chair stand 21 repetitions). Load–velocity profiling revealed unusually slow minimal-velocity thresholds, causing standard group-based equations to underestimate measured one-repetition maximum by 11–28%, while load–power curves demonstrated high peak power at approximately 57% of one-repetition maximum in both exercises. Psychological assessments indicated high self-efficacy (35/40) and self-esteem (34/40), and qualitative themes highlighted strength as a foundation for resilience, emotional regulation, unwavering adherence, empowered body image, and training as a central life identity. Together, these integrated findings suggest that long-term, supervised HIRT may support a virtuous cycle in later life in which exceptional strength, power, favorable body composition, and functional independence are tightly linked with robust psychological resources and sustained adherence.

## Introduction

Population aging presents unprecedented challenges for health and social-care systems worldwide. The number of adults aged 65 and older is projected to reach 1.5 billion by 2050, more than double the 727 million in 2020, due to increasing life expectancy and declining fertility rates (United Nations, 2020). Aging is commonly associated with sarcopenia, a progressive loss of skeletal muscle mass Wand metabolic function that contributes to reduced mobility, increased fall risk, and diminished quality of life (Larsson et al., 2019; Wilkinson et al., 2018). Maintaining muscular strength, balance, and coordination is therefore essential for preserving independence and quality of life in older adults (Brogno, 2025).​

Among available interventions, high-intensity resistance training (HIRT) has emerged as a leading strategy for counteracting age-related physical decline (Izquierdo et al., 2025; Fragala et al., 2019). Evidence shows that HIRT can preserve and improve muscle mass, strength, and power; enhance functional capacity; reduce fall incidence; and promote autonomy and quality of life, including in older adults with frailty, sarcopenia, osteoporosis, or mobility limitations and in institutional or hospital settings (Izquierdo et al., 2025; Fragala et al., 2019). HIRT is often feasible, and sometimes preferable, when aerobic training is limited by multimorbidity or functional impairment (Papa et al., 2017). Beyond physical benefits, resistance training has been linked to better psychosocial wellbeing, including higher self-esteem and self-efficacy, more positive body image and social functioning, and reduced symptoms of anxiety and depression, contributing to greater overall life satisfaction (Cunha et al., 2024; Churchill et al., 2022; McAuley et al., 2011; Marcos-Pardo et al., 2019a). These findings support the integration of personalized resistance-training regimens into routine geriatric care as a core component of strategies to preserve health span and mitigate age-related disability (Izquierdo et al., 2025; Fragala et al., 2019).

In parallel, research in exercise psychology has increasingly examined the lived experiences of aging and physical activity, highlighting how older adults make sense of, and adapt to, movement-based interventions (Ambrens et al., 2023; González-Calvo et al., 2025). However, studies that integrate objective physiological data with detailed psychological assessment and qualitative accounts of meaning, identity, and adherence remain scarce. Related case studies in elite female master athletes have provided rich physiological profiles of maximal strength, aerobic capacity, and in some cases muscle morphology (Cattagni et al., 2020; Hooren et al., 2022; Saillant et al., 2023; Fuchs et al., 2024; Colosio et al., 2025). Yet the psychological dimensions of these athletes have received limited attention: only Saillant et al. (Saillant et al., 2023) included psychological outcomes, and assessment there was restricted to generic measures of quality of life and depression, without examining constructs such as self-efficacy or self-esteem or incorporating qualitative interviews. Furthermore, the existing case-study literature focuses on competitive master athletes (Cattagni et al., 2020; Hooren et al., 2022; Fuchs et al., 2024), leaving an important gap regarding non-competitive but highly trained older exercisers who show comparable dedication and physiological adaptation outside of formal sport environments.

To address these gaps and broaden the literature beyond elite athletic populations, this N = 1 mixed-methods study presents an upper-bound example of adaptation in a highly trained older adult rather than a typical aging trajectory. It is intended to illustrate the ceiling of what may be possible under long-term, supervised HIRT and to generate practice-relevant hypotheses about mechanisms and determinants of successful aging that can inform program design and service delivery for older adults. This single-case convergent mixed-methods design integrates detailed quantitative assessments of body composition, maximal strength, functional performance, and load-velocity (L-V) and load-power (L-P) relationships with standardized self-efficacy and self-esteem questionnaires and a semi-structured interview exploring training, identity, and societal views of aging and gender. The methodological approach aligns with Halperin’s argument that single cases can deepen understanding of the physiological and psychological processes of unique individuals and act as a bridge between science and applied practice (Halperin, 2018). Recent WHO work on *vitality capacity* emphasizes the need to assess multisystem resources that support functional ability and healthy longevity, integrating physical, metabolic, and psychosocial dimensions. In this context, detailed N = 1 mixed-methods designs can provide rich, person-level profiles of vitality capacity, illustrating how long-term resistance training shapes interconnected physiological and psychological domains in later life (Bautmans et al., 2022). By combining these data sources, this study seeks to identify modifiable levers such as training structure, supervision, psychological strategies, and lifestyle context that may help clinicians, trainers, and community programs support aging strong in later life. Accordingly, this N = 1 mixed-methods study aimed to (i) characterize an upper-bound example of aging strong after 8 years of supervised HIRT in a 71-year-old woman and (ii) develop a conceptual virtuous-cycle model that integrates physiological, psychological, and qualitative data.

## Methods

This investigation used a convergent N = 1 mixed-methods design in which quantitative and qualitative data were collected within the same assessment period. N = 1 designs are increasingly recognized as useful for examining individual-level mechanisms, testing intervention feasibility, and identifying upper-bound adaptations that can inform hypotheses for larger exercise-and-aging trials. In musculoskeletal aging, detailed single-participant studies are particularly valuable because exceptional responders may display physiological and psychological adaptation patterns that are masked in group averages. Accordingly, the present design aimed to provide an in-depth, hypothesis-generating exemplar of long-term HIRT in later life, using integrated quantitative and qualitative data to illuminate potential mechanisms and practice-relevant levers.

### Participant

The participant was a 71-year-old woman with over 2 decades of regular exercise experience, including eight consecutive years of supervised HIRT. Ethical approval was obtained from an institutional review board, and she provided written informed consent after receiving full information about the study and her right to withdraw at any time. She was purposively selected as an upper-bound exemplar based on her long-term supervised HIRT, consistently exceptional observed performance, and willingness to participate, rather than to represent typical older adults.

### Physical activity history

The participant’s youth was marked by scoliosis and chronic back pain, and she was discouraged from sports by physical education teachers, which fostered negative attitudes toward physical activity and limited her participation in organized sport during childhood and adolescence. At age 45 she engaged with structured exercise through hydrotherapy and Ashtanga yoga, developing movement control and flexibility and experiencing a turning point as she realized she could perform tasks she had long been told she could not, building competence and emotional satisfaction. At age 50 she began aerobic training and was introduced to strength exercise under a personal trainer at low to moderate intensities, but several trainers were reluctant to assign heavier loads because of her age and back history, limiting her progression at that time.

### High-intensity resistance training phase

Under the supervision of one of the authors (a certified personal trainer), the participant progressed to high-intensity resistance training at age 63, completing three structured sessions per week (∼60 min) using an upper-body, lower-body plus arms, and full-body split. A representative weekly training structure, including exercise selection, set and repetition ranges, and approximate weekly loading per muscle group, is summarized in Table 1. Training followed ACSM resistance-training progression principles for healthy adults (Currier et al., 2026; American College of Sports Medicine, 2025), with exercises performed at high effort (typically close to muscular fatigue or momentary failure) and loads progressed gradually over time while adjusting for fatigue to balance progressive overload with recovery and injury risk. Despite occasional knee or shoulder discomfort, she maintained full adherence, modifying exercises when necessary (e.g., changing seat angle, adjusting range of motion, or reducing loads).

**TABLE 1:** Representative weekly high-intensity resistance training structure.

Training component	Day 1 upper body	Day 2 lower body + arms	Day 3 full body	Weekly sets per muscle group
Main focus	Upper-body pushing and pulling, with supplemental arm and shoulder work	Lower-body strength with additional arm work	Combined upper- and lower-body training	Upper body (total 45): Chest ∼15 Back ∼15 Shoulders ∼3 Biceps ∼6 Triceps ∼6 Lower body (total 27): Quadriceps ∼15 Hamstrings ∼6 Glutes and erector spine ∼6
Exercises (examples)	Barbell bench press; weighted push-ups; cable chest fly; pull-ups (wide/supinated); single-arm cable row; dumbbell lateral raises; incline dumbbell curls; cable triceps extensions	45° leg press; machine knee extension; Bulgarian split squat; seated leg curl; roman-chair back extensions; dumbbell curls; cable triceps	Barbell bench press; cable chest fly; pull-ups; single-arm cable row; 45° leg press; machine knee extension; seated leg curl; roman-chair back extension; dumbbell curls; cable triceps extension	
Sets × reps (typical)	3 × 5–8 multi-joint exercises 3 × 8–12 single joint exercise	3 × 4–8 multi-joint exercises 3 × 8–12 single joint exercise	3 × 5–8 multi-joint exercises 3 × 8–12 single joint exercise	

The table presents a representative weekly structure of the participant’s high-intensity resistance training (HIRT), performed three times per week (∼60 min; upper body, lower body plus arms, full body). Exercises were typically performed for 3 sets of ∼5–12 repetitions with ∼2-min rests, close to muscular fatigue or momentary failure, with gradual load progression in line with ACSM, resistance-training guidelines (Currier et al., 2026; American College of Sports Medicine, 2025). To balance progressive overload with injury risk, exercise selection, loads, and order were periodically adjusted in response to fatigue and minor discomfort. Weekly loading per muscle group reflects direct work for primary movers based on this typical structure and does not include additional indirect loading from compound exercises. Accordingly, values should be interpreted as approximate rather than fixed.

Complementing the physical aspect, the participant was consistently encouraged to use motivational self-talk (i.e., self-directed statements spoken aloud or internally) during high-intensity efforts to boost persistence and achieve maximal output, and to use instructional self-talk when learning new exercises or refining technique. This approach aligns with sport psychology research showing that motivational self-talk enhances effort and performance under high loads, whereas instructional self-talk is particularly effective for skill acquisition and technical improvement (Bellomo et al., 2020).

### Lifestyle factors

The participant maintained high daily activity (∼8.5 km brisk walking) and a balanced, protein-rich diet (yogurt, eggs, protein powder, fish, vegetables, dairy; minimal fats and sugars), maintaining stable body weight (∼50 kg) since early adulthood. During the COVID-19 lockdowns (2020–2021), she equipped a comprehensive home gym (Smith machine, cable cross, leg press, bench press, leg extension machine, both lying and seated leg curl machines, adjustable free weights, and accessories), allowing her to maintain uninterrupted training and progression despite public facility closures.​

### Psychological assessment

To assess psychological characteristics relevant to successful aging, the participant completed two validated self-report questionnaires.​

New General Self-Efficacy Scale (NGSE) (Chen et al., 2001), The eight-item scale measures global confidence in handling various challenges using a five-point Likert scale (1 = “strongly disagree” to 5 = “strongly agree”). The NGSE shows excellent psychometric properties and serves as a refined alternative to earlier self-efficacy measures (Schwarzer and Jerusalem, 2012). Higher scores indicate greater self-efficacy.​

Rosenberg Self-Esteem Scale (RSES) (Rosenberg, 1965). This widely used 10-item scale measures overall self-worth using a 4-point Likert scale (1 = “strongly agree” to 4 = “strongly disagree”). The RSES maintains excellent internal consistency (Cronbach’s α = .82-.93), strong temporal stability (r = .85 over 2 weeks), and convergent validity with related constructs (Schmitt and Allik, 2005; Sinclair et al., 2010). Higher scores indicate greater self-esteem.​

### Qualitative measurement

A semi-structured interview was conducted in the participant’s home to explore lived experiences related to long-term HIRT. The interview protocol was adapted from Marrella (Marrella, 2024) and addressed five primary domains: first, motivation and adherence; second, aging identity and perceptions; third, physical self-concept and body image; fourth, perceived benefits of training; and fifth, social and relational factors. Only the participant and interviewer were present, and the interview was conducted in the participant’s native language in a quiet room of her home to maximize comfort and openness. The interview was audio-recorded with consent and transcribed verbatim. Qualitative data were analyzed using reflexive thematic analysis (Braun and Clarke, 2006). All coding and theme development were conducted manually. The primary analyst (N.M.), who had been the participant’s personal trainer for 8 years and holds a master’s degree in sport and exercise psychology with qualitative interviewing experience, conducted initial coding by identifying meaning units and emergent patterns, which informed overarching themes. To mitigate potential bias associated with the dual trainer–researcher role, preliminary themes and reflexive notes on assumptions and expectations were reviewed and discussed with a co-author not involved in the participant’s training. Because this single-participant N = 1 design prioritized depth rather than saturation, adequacy was judged by the richness and completeness of the in-depth interview rather than by conventional saturation criteria.

### Quantitative assessment of physical function

To assess the participant’s physical capabilities, we measured performance in three sub-domains: first, maximal strength and muscular endurance; second, functional performance, L-V and L-P characteristics; and third, body composition. All measurements were conducted across three sessions over 1 week, with ≥48 h between sessions to minimize residual fatigue. Session 1: maximal strength testing one-repetition maximum (1RM; 45° leg press, barbell bench press) and push-up-to-failure. Session 2: functional performance tests (SPPB, TUG, 30-s chair stand) and L-V profiling (45° leg press, barbell bench press). Session 3: dual-energy X-ray absorptiometry (DXA) body composition assessment. All tests were scheduled at similar times of day (±2 h) to standardize testing conditions across sessions. Additionally, all planned assessments were completed without missing data; therefore, no imputation procedures were required.​

Pre-testing standardization. The participant followed standardized protocols: 48-h exercise abstention, maintained hydration, avoided caffeine and consumed a light meal (2–3 h prior), wore appropriate attire, and obtained adequate sleep (∼7 h) (McGowan et al., 2015). Environmental conditions were maintained at comfortable room temperature. Certified personnel supervised all sessions ensuring safety and proper technique. A standardized warm-up preceded all physical assessments: 5 min low-intensity cardiovascular activity (treadmill) at 50%–60% HR_max_ or rate of perceived effort 3-4 followed by dynamic stretching and progressive movement preparation (McGowan et al., 2015).

### Maximal strength testing

All 1RM testing followed established guidelines with proven reliability (Grgic et al., 2020). Following general warm-up, specific preparation included: 5-8 repetitions at 40%–60% estimated 1RM (2-min rest), then after, 3-5 repetitions at 60%–80% estimated 1RM (3-min rest), and lastly an initial 1RM attempt at 90%–105% estimated 1RM, with subsequent attempts using 2.5%–5% increments/decrements and 3–5 min rest between trials. The 1RM represents maximum resistance completed with proper single execution (Amarante et al., 2013). 1RM testing was complete when the participant and the examiner agreed that no additional single repetition could be safely completed. During all strength tests, motivational self-talk was encouraged alongside verbal encouragement to maximize physical output. Both strategies have been shown to enhance strength performance and facilitate maximal effort (Bellomo et al., 2020).

Equipment and procedures. Leg press assessments used a 45° GymTech leg press machine. The participant was positioned with feet shoulder-width apart at mid-platform, knees aligned with toes. Range of motion was standardized at 90° knee flexion (verified via goniometer) to full extension. Barbell bench press used a standard Olympic setup with established procedures. The participant lay supine with feet flat, maintaining five points of contact (head, upper back, buttocks, left foot, right foot). The bar was lowered until lightly touching the chest at mammilla line, then pressed to full elbow extension.​

### Functional performance assessment

Short Physical Performance Battery. The SPPB comprises three components scored 0–4 each (maximum 12): first, standing balance (side-by-side, semi-tandem, and tandem positions held 10 s each); second, a 4-meter walk at usual pace (average of two trials); and third, five repetitions of chair stands (completion time) (Guralnik et al., 1994). All procedures followed standardized SPPB protocols with one practice trial for each component.​

Timed Up and Go. Following established procedures (Podsiadlo and Richardson, 1991), the participant rose from a standard chair (43 cm height), walked 3 meters at a comfortable pace, turned around a cone, returned, and sat down. One practice trial ensured familiarization. Two trials were performed with 1-min inter-trial rest, with the faster time recorded (Christopher et al., 2021).

30-s chair stand test. Following standardized protocols (Jones et al., 1999), the participant completed as many chair stands as possible in 30 s from a standardized chair (same chair for all measurements). Starting position was seated in the center of the chair, back straight, feet flat shoulder-width apart, arms crossed over the chest. Each repetition required full hip and knee extension to standing, followed by a return to the seated position; only complete repetitions were counted.​

Push-ups to muscular failure. After a brief warm-up (3-min fast-paced treadmill walk plus dynamic upper-body movements and light resistance-band exercises (Adams et al., 2022)). The participant performed consecutive push-ups to voluntary exhaustion or technique failure. Each repetition required straight body alignment, lowering the chest to a 5-cm foam pad, and returning to full elbow extension; repetitions with improper form or incomplete range of motion were not counted.

### L-V and L-P profiling

Measurement protocol. L-V and L-P relationships were assessed using a linear position transducer (GymAware PowerTool, Kinetic Performance Technology, Canberra, Australia) sampling at 50 Hz based on validated protocols for older adults (Alcazar et al., 2017). After warm-up at 30%–50% estimated 1RM, the participant performed two maximal-velocity repetitions across progressive loads (∼40–80% 1RM; 2.5-kg increments for the bench press, 10-kg increments for the leg press), with 60–120-s rests adjusted based on repetition velocity to ensure adequate recovery (Alcazar et al., 2017).

Data modeling. The L-V relationship was modeled using simple linear regression: Velocity = Intercept + (Slope × Load). The predicted one-repetition maximum (1RM_pred) was estimated by extrapolating the regression line to a fixed minimal-velocity threshold (MVT; V_1RM = 0.15 m·s^-1^ for the barbell bench press and 0.20 m·s^-1^ for the 45° leg press), using values slightly below the means reported by Marcos-Pardo et al. (2019a). The L-P relationship was modeled using a second-order polynomial, Power = a + b·Load + c·Load^2^, from which the optimal load (L_opt) and maximal mean power (P_max) were calculated as the vertex of the parabola (Alcazar et al., 2018). All regression analyses and goodness-of-fit (*R* ^2^) statistics were obtained using GraphPad Prism (version 10, GraphPad Software, San Diego, CA, United States).

### Body composition measurements

Dual-energy X-ray absorptiometry. Whole-body and regional body composition were assessed via DXA (Hologic Discovery, software version 13.5) following standardized guidelines (Kendler et al., 2013). Scans were performed in the morning after a 12-h overnight fast and ≥24 h post-exercise. The participant avoided calcium supplements (24 h prior) and wore metal-free clothing.​

Derived variables. From DXA output, the following were calculated: A: fat-free mass (FFM) = lean mass + bone mineral content, B: appendicular lean mass (ALM) = sum of arm and leg lean mass, C: appendicular lean mass index (ALMi) = ALM/height^2^, and D: relative ALM (%ALM) = (ALM/body mass) × 100. Android and gynoid fat percentages and android/gynoid ratio characterized regional fat distribution.​

### Data integration

Quantitative indicators of body composition, strength, neuromuscular function, functional performance, and questionnaire scores were compared side-by-side with emergent qualitative themes, and this integration informed the development of the conceptual “virtuous-cycle” model of aging strong with HIRT.​

## Results

### Participant characteristics and body composition

At assessment, the participant was 71.2 years old with height 162 cm, body mass 50.0 kg, and BMI 19.1 kg·m^-2^. DXA analysis revealed highly desirable body composition: whole-body fat 15.1% (7,416 g), android fat 10.5%, gynoid fat 26.5% (android/gynoid ratio 0.39), FFM 43,494 g, ALMi 10.33 kg·m^-2^, %ALM 53.9%, and bone mineral content 1,761 g. In most aspects, these values substantially exceed typical age-matched norms based on DXA reference data for older women (Ofenheimer et al., 2020; Kelly et al., 2009) (see Table 2 for comparative reference data). These data illustrate a lean, muscular phenotype that is uncommon in women in their seventies and is consistent with a low risk of sarcopenia and metabolic complications in clinical practice.

**TABLE 2:** Physiological characteristics and performance outcomes compared with normative data for older women.

Variable	Participant	Normative/References values	Interpretation
Anthropometrics and body composition			
Age (years)	71.2	—	—
Height (cm)	162	—	—
Body mass (kg)	50.0	—	—
BMI (kg·m^-2^)	19.1	∼27^d^	Markedly lean physique
Whole-body fat (%)	15.1	∼40–41% (age ∼70)^c^	Exceptionally low adiposity
Fat-free mass (kg)	43.49	∼40.0^c^ (age matched)	High relative lean mass
ALMi (kg·m^-2^)	10.33	∼6.5 (age ∼70)^c^	Far exceeds sarcopenia cutoff; excellent muscularity
%ALM (% of body mass)	53.9	∼35–40%	Far greater proportion of mass as limb lean tissue than age-matched norms
Bone mineral content (g)	1,761	1879(g)	Similar to age-matched reference
Android/Gynoid fat ratio	0.39	0.9–1.0^d^	Substantially lower central adiposity than typical older women
Maximal strength			
Bench press 1RM (kg)	52.5 (1.05× BW)	22.3 kg, SD ≈ 4.7 kg^i^	Above 90th percentile even compared to competitive athletes
Leg press 1RM (kg)	192.7 (3.85× BW)	114.6 kg SD ≈ 15.9 kg^i^	Relative strength approximately 2.3 times reference values
Muscle power			
Peak mean bench-presses power (W·kg^-1^)	3.28	1.0–1.8 (typical older women)^a^	Above average; high functional capacity
Peak mean leg-press power (W·kg^-1^)	4.30	2.1 (low functional threshold)^b^ 1.5–2.5 typical older women^a^	Exceptional; ∼2× typical range; far exceeds disability risk thresholds
Muscular performance			
Push-ups (reps)	27	≥17 = excellent^h^	Exceeds 30–39-year-old norms – excellent upper extremity muscular endurance
30s chair stand (reps)	21	10–11^f^	Excellent lower-body muscular endurance
Timed up and go (s)	6.08	8.5^e^	Exceptional mobility – low fall risk
SPPB (0–12 scale)	12	8–9^g^	Maximal functional ability – low fragility risk

^a^ Power reference values are approximate and derived from studies using different equipment and movement tasks; comparisons should be interpreted cautiously.

^b^ Alcazar et al.(2021b) clinically validated threshold for low functional capacity in sit-to-stand; leg-press power values shown here are considerably higher, suggesting protective reserve for activities of daily living.

^c^ Kelly et al. (2009) Reference values are for 50th percentile, NHANES, database; FFM, is derived from DXA, as LM + BMC, for 50th percentile.

^d^ Ofenheimer et al. (2020) BMI, reference mean age group 70-<82. Android/Gynoid fat ratio, age group 70-<82, reference values 50th percentile.

^e^ Podsiadlo and Richardson (1991); Mahato et al. (2004) TUG, values compared with age matched 50th percentile norms.

^f^ Jones et al. (1999) 30s STS, values age matched (70–74) present as mean, typically above average is 12–17.

^g^ Ramírez-Vélez et al. (2020) values are 50th percentile women 70–79.

^h^ ACSM’s Guidelines for Exercise Testing and Prescription (12th ed.). American College of Sports Medicine (2025) women aged 60–69.

^i^ Marcos-Pardo et al. (2019b) provide age-matched bench-press and leg-press 1RM, values for community-dwelling older women, whereas van den Hoek et al. (2024) report age-matched reference values for elite powerlifters.

### Maximal strength and functional performance

Maximal strength. The participant’s bench press 1RM performance reached 52.5 kg (1.05× body weight), exceeding the 90th percentile for competitive older-adult women aged 60–79 years reported in a large-scale analysis of 21,524 powerlifters by van den Hoek et al., 2024 (van den Hoek et al., 2024), where typical strength-to-body-weight ratios cluster around 0.6–0.7. Her leg press 1RM performance reached 192.7 kg (3.85× body weight), which is also exceptional and aligns with values documented in a case study of a master-level powerlifter champion of similar age (Fuchs et al., 2024), indicating a remarkably high lower-body strength capacity for an older adult.

Muscular endurance. Push-ups to muscular failure testing yielded 27 repetitions, surpassing the American College of Sports Medicine “Excellent” threshold for women aged 60–69 years (≥17 repetitions) and aligning with the “Excellent” threshold for women aged 30–39 years (American College of Sports Medicine, 2025) (Table 2). This upper-body endurance profile indicates that she functions, by this metric, more like a much younger adult than a typical woman in her eighth decade.

Functional performance. The 30-s chair stand test yielded 21 repetitions, TUG was 6.08 s, and SPPB achieved a perfect score of 12/12. These results reflect excellent mobility, a high level of muscular endurance, low fall risk, and minimal disability risk (Guralnik et al., 1994; de Fátima Ribeiro Silva et al., 2021) (Table 2). Clinically, this constellation would generally be interpreted as indicating very low current risk of mobility limitation and high likelihood of independent community living.

### Psychological assessment

Self-report questionnaires indicated high general self-efficacy (35/40) and high self-esteem (34/40), reflecting strong confidence and self-worth (see Table 3). These scores are consistent with a resilient psychological profile, which may support adherence to demanding resistance-training regimens in older adulthood.

**TABLE 3:** Psychological assessment results (New General Self-Efficacy Scale and Rosenberg Self-Esteem Scale) with normative values.

Measure	Scale range	Irit score	Normative value	Percentile/Interpretation
General self-efficacy (NGSE)	8–40	35	31.6 ± 5.7	High level of self-efficacy
Self-esteem (rosenberg)	10–40	34	29.9 ± 5.6	High level of self-esteem

Normative values are presented as mean (SD).

Self-efficacy norms from Gobeille et al., 2024 based on adults aged 55–94.

Self-esteem norms from Ryszewska-Łabędzka et al., 2022 based on adults over 60 years of age.

### L-V and L-P profiling

**FIGURE 1:**
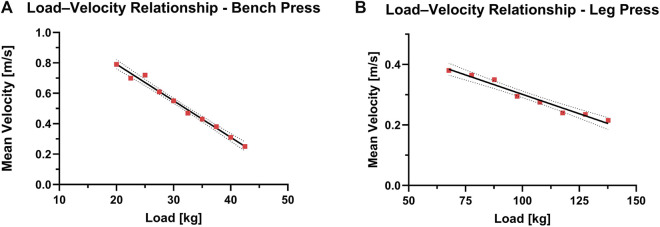
Load-velocity relationships in the barbell bench press and 45° leg press. Each point represents mean concentric velocity at each submaximal load for the barbell bench press (panel **(A)**) and 45° leg press (panel **(B)**), with solid lines indicating the fitted linear regressions and dotted lines indicating the 95% confidence bands.

**FIGURE 2:**
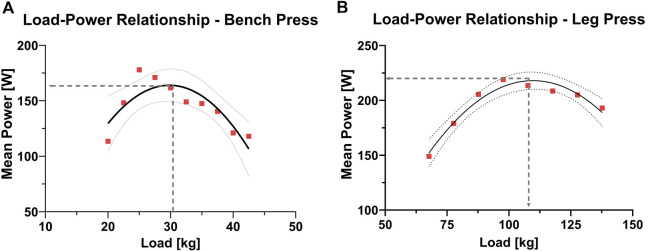
Load-power relationships in the barbell bench press and 45° leg press. Red squares show mean concentric power at each submaximal load for the bench press (panel **(A)**) and 45° leg press (panel **(B)**). Solid curves represent quadratic load–power fits, with dotted lines denoting 95% confidence bands and grey dashed lines marking the load corresponding to peak mean power in each exercise.

The barbell bench press and 45° leg press both demonstrated strong linear L-V relationships across the tested submaximal load ranges (20–45 kg for the bench press; 67.7–137.7 kg for the leg press), with coefficients of determination of *R* ^2^ = 0.987 and *R* ^2^ = 0.968, respectively (see Figure 1). When conventional MVTs were applied (0.15 m·s^-1^ for the bench press; 0.20 m·s^-1^ for the leg press), the predicted 1RM values (46.7 kg and 139.5 kg) underestimated the directly measured 1RM (52.5 kg and 192.7 kg) by 11% and 28%, respectively; the corresponding extrapolated V_1_RM values (0.012 and 0.064 m·s^-1^) were near isometric. Both exercises displayed clear parabolic L-P relationships (bench press *R* ^2^ = 0.72; leg press *R* ^2^ = 0.95; see Figure 2). For the bench press, the quadratic model yielded a mean power of 164 W at an optimal load of 29.9 kg (57% 1RM), whereas for the 45° leg press it yielded 215 W at 110 kg (57% 1RM). Collectively, these findings suggest that standard group-based MVTs derived from less-trained older women may underestimate true capabilities in highly trained older lifters, highlighting the need for individualized L-V profiling when prescribing and monitoring HIRT.

### Qualitative findings

A semi-structured interview (∼60 min) and reflexive thematic analysis identified eight primary themes reflecting long-term HIRT experiences in older adulthood (Table 4). These interconnected themes show how sustained resistance training shaped the participant’s psychological, physical, and social functioning. Beyond serving as a health-promoting behavior, resistance training played a deep and multifaceted role in her life and acted as a central organizing principle rather than merely a form of physical activity. Physical strength emerged as a foundation for mental resilience. Training also functioned as a key mechanism for emotional regulation and coping, supporting cognitive clarity and rational decision-making during periods of stress and loss. Unwavering adherence was described as a defining characteristic, with persistence framed as a non-negotiable part of identity rather than a behavior dependent on motivation or circumstances. Resistance training further contributed to an empowered body image, marked by pride in muscularity, rejection of age-related criticism, and a redefinition of femininity in later life. The trainer–trainee relationship was experienced as a critical source of emotional support, relatedness, and accountability. Training was also perceived as a way to preserve functionality and vitality, enhance mobility and confidence, and protect against injury. Beyond the individual level, the participant described a strong role-modeling effect, as her training influenced family members’ health behaviors and challenged intergenerational stereotypes about aging.

**TABLE 4:** Summary of themes from reflexive thematic analysis of the semi-structured interview.

Main theme	Subthemes	Representative quote	Interpretation
1. Mental resilience through physical strength	Integration of physical–psychological domains; transfer to life	*“The training gives me independence and control… I’m much stronger, also mentally.”*	Physical strength fosters psychological resilience and autonomy
2. Emotional regulation & coping	Grief, stress regulation, cognitive clarity	*“After training, I can see things more from the side… I become more balanced and rational.”*	Exercise functions as a regulatory and stabilizing mechanism
3. Unwavering adherence (“never giving up”)	Injury adaptation, persistence, non-negotiable training	*“There’s no chance I cancel a workout.”*	Adherence rooted in identity and resilience
4. Empowered body image	Pride in strength, rejecting criticism	*“My grandchildren ask me to flex my biceps.”*	Positive redefinition of aging femininity
5. Trainer relationship as critical support	Emotional bond, feedback dependence	*“Without a trainer, many people quit.”*	Supervision satisfies relatedness and enhances adherence
6. Functionality & vitality protection	Fall prevention, mobility, cognitive clarity	*“I fell hard… The muscles and bone strength protected me.”*	Training preserves independence and confidence
7. Role modeling & intergenerational impact	Family inspiration, stereotype challenge	*“My husband lost* 10 kg*… It’s a lot because of me.”*	Personal empowerment ripples through family systems
8. Training as identity integration	Centrality, mood enhancement	*“It’s part of me… part of my life.”*	Exercise as integrated self-definition and emotional anchor

## Discussion

This single-case investigation illustrates the longitudinal benefits of supervised HIRT in a 71-year-old woman whose 8 years of training resulted in an unusually high physiological and psychological profile. Her outcomes in body composition, maximal strength, neuromuscular profiling, and psychological resources not only exceeded age-matched reference values for older women but, in several indices, lay at or beyond the upper end of distributions reported for community-dwelling and trained older adults (Ofenheimer et al., 2020; Kelly et al., 2009; Parr et al., 2024). Her bench-press 1RM relative to body mass lies around the 90th percentile of international powerlifting standards for women in her age band, and her leg-press 1RM is comparable to values reported in a master-level powerlifting champion of similar age, indicating an extreme, highly trained phenotype (Fuchs et al., 2024; van den Hoek et al., 2024). These findings should therefore be interpreted as an upper boundary of adaptation rather than realistic clinical targets for most community-dwelling older adults. Although her performance aligns with, and in some respects exceeds, values reported in master-level powerlifters, she is not a competitive athlete but a non-elite, community-dwelling older woman who only took up structured resistance training in midlife. This distinction is important: despite being an exceptional responder, she represents the kind of person many clinicians and exercise professionals routinely encounter, rather than a lifelong elite performer.

Her body composition and strength profile reinforce this interpretation. Low body fat (15.1%), high ALMi (10.33 kg·m^-2^), and elevated %ALM (53.9%) clearly exceed population norms for women in their seventies and are well above sarcopenia thresholds, a constellation associated with lower cardiometabolic risk, reduced disability, and decreased all-cause mortality (Ofenheimer et al., 2020; Kelly et al., 2009; Cruz-Jentoft et al., 2019; Tyrovolas et al., 2020). Although cardiometabolic outcomes were not directly measured in this case, maintaining high lower-body strength and lean mass in later life may indirectly support cardiometabolic health. Greater strength might reduce the perceived effort of walking and everyday mobility tasks, making movement easier and more comfortable, which in turn may facilitate more spontaneous free-living physical activity and help sustain a healthy body weight and cardiometabolic profile (Cenko et al., 2021). In addition, resistance training induced increases in muscle mass and strength have been linked to improvements in insulin sensitivity, glucose homeostasis, and several cardiometabolic risk factors, underscoring the broader metabolic value of maintaining skeletal muscle in older adults (Galancho-Reina et al., 2019; Zaleski et al., 2016). Emerging evidence also indicates that resistance exercise acutely modulates myokines and neurotrophic factors, such as IL-6, IL-15, IGF-1, BDNF, and VEGF, which may contribute to favorable immunometabolic and neurovascular adaptations with potential relevance for long-term cardiometabolic and functional health (Cheng et al., 2022; Zunner et al., 2022). When expressed relative to body mass, her maximal strength (bench press 1.05× body weight; leg press 3.85× body weight) is markedly higher than values reported for community-dwelling older women in bench-press and leg-press testing, and falls within the upper percentile range of age- and weight-graded bench-press standards for competitive female powerlifters (van den Hoek et al., 2024). Together with evidence linking higher muscle mass and strength to preserved physical independence and lower risk of disability and falls, these metrics underscore the potential for long-term, individualized HIRT to support an independent, active life in later adulthood, at least for selected older adults who can tolerate and access this type of training (Parr et al., 2024; Mahato et al., 2024).​ From an applied standpoint, such strength levels are far beyond those typically observed in community-dwelling older women and provide a substantial reserve for daily-living and protective responses (e.g., recovering from perturbations) (Saillant et al., 2023; Leenders et al., 2013). At the same time, HIRT is not universally appropriate. Recovery from resistance exercise is often slower and more variable in older adults, so excessive volume or load can overburden recovery capacity and increase injury risk, particularly in those with multimorbidity or low baseline fitness. This case therefore underscores the need for careful screening, individualized progression, and close supervision when implementing higher-intensity programs in later life Table 1 (Hayes et al., 2023).

In line with body composition and maximal strength results, flawless functional performance (Short Physical Performance Battery 12/12, Timed Up and Go 6.08 s, 30-s chair stand 21 repetitions) indicates exceptional neuromuscular efficiency and independence far surpassing her chronological age (Guralnik et al., 1994; Podsiadlo and Richardson, 1991; Mahato et al., 2024). From a clinical perspective, the combination of high maximal strength, muscular endurance, and functional performance, all of which are responsive to HIRT, suggests a substantial reserve capacity for daily tasks and rapid responses to perturbations (for example, slips or trips), a key target for geriatric exercise interventions (Izquierdo et al., 2025; Cruz-Jentoft et al., 2019).

Strong linear L-V relationships were observed for both exercises, and her extrapolated V_1_RM values were substantially lower than the 0.17–0.21 m·s^-1^ 1RM velocities reported for older women in group-based L-V studies (Marcos-Pardo et al., 2019b). When conventional group-derived MVTs (0.15 m·s^-1^ for the bench press; 0.20 m·s^-1^ for the leg press) were applied to her individual L-V profiles, they underestimated her directly measured 1RM by 11%–28%, a novel finding in a highly trained older adult (García-Ramos, 2023). Together with evidence that reference MVTs and group-based equations can bias 1RM estimates compared with individualized approaches, our findings suggest that fixed MVTs are unsuitable for high-responder older lifters and that individualized L-V profiling is preferable. This systematic underestimation of 1RM, despite excellent L-V model fits, reinforces the need to base prescriptions on individual profiles when monitoring neuromuscular adaptations in older adults engaged in long-term HIRT (Alcazar et al., 2018; García-Ramos, 2023). Her unusually slow V_1_RM values likely reflect both methodological and individual factors, including equipment characteristics, a deliberately controlled lifting strategy near failure, long-term progressive HIRT, and a high willingness to exert effort under fatigue; however, these mechanisms remain hypothesis-generating rather than directly measured.

Both exercises showed clear quadratic L-P relationships, with mean peak power outputs of 164 W in the barbell bench press and 215 W in the 45° leg press, each occurring at approximately 57% of 1RM. When normalized to body mass (3.28 W·kg^-1^ and 4.30 W·kg^-1^, respectively), these values are consistent with exceptionally high neuromuscular performance for a 71-year-old woman and exceed leg-press and chest-press power norms reported for community-dwelling older women tested on pneumatic machines, where smoother resistance would typically favor higher power outputs (Parr et al., 2024). For context, low functional capacity in older adults has been associated with relative sit-to-stand power values around or below ∼2.0–2.1 W·kg^-1^, thresholds linked to frailty, mobility limitations, and poorer quality of life (Alcazar et al., 2021a; Alcazar et al., 2021b). The participant’s values suggest a large buffer above power-related risk thresholds, aligning with her excellent functional test scores and illustrating how HIRT can enhance power-based indicators of healthy aging. Long-term resistance training in older adults can improve rate of force development and muscular power, which relate more strongly than maximal strength or muscle mass to mobility, balance, and independence, so her elevated limb-power profile likely confers protective benefits for functional autonomy and health beyond those expected from strength or body-composition indices alone (Larsson et al., 2019; Izquierdo et al., 2025; Fragala et al., 2019; Papa et al., 2017; Cruz-Jentoft et al., 2019). Such power and strength levels create a large physiologic reserve above the threshold required for daily tasks, which might reduce perceived effort during common activities and may attenuate fatigue in response to both physical and psychosocial stressors in everyday life. Much of the longevity and aging literature has emphasized maximal aerobic capacity (VO_2_max) as a key marker of prognosis. However, for community-dwelling older adults, the ability to generate high relative muscular power and strength at submaximal intensities may be more directly relevant to the demands of activities of daily living and fall avoidance, as illustrated by the present case.

Psychological assessment showed high general self-efficacy (35/40) and self-esteem (34/40), reflecting strong confidence and self-worth at an age when these resources often decline (Robins et al., 2002; Wagner et al., 2015). Such sustained high scores contrast with typical late-life patterns of flattening or decrease in self-esteem and self-efficacy, even when cultural differences are considered (Ogihara and Kusumi, 2020). In the interview, she described training as a key source of independence and control *(“the training gives me independence and control … I am much stronger, also mentally”)*, illustrating how physical competence reinforced intrinsic motivation and adherence. This pattern fits self-efficacy theory, in which repeated mastery experiences and supportive feedback strengthen confidence and long-term engagement in demanding activity (Bandura, 1997). These experiences show a reciprocal reinforcement cycle: physical competence may boost intrinsic motivation, which in turn supports adherence to training. This pattern of high perceived competence and sustained adherence aligns directly with established psychological frameworks of self-efficacy and Self-Determination Theory. In this case, training was experienced as autonomous and competence-enhancing, and the close trainer–trainee relationship fulfilled the need for relatedness (Bandura, 1997; Ryan and Deci, 2000).​

**FIGURE 3:**
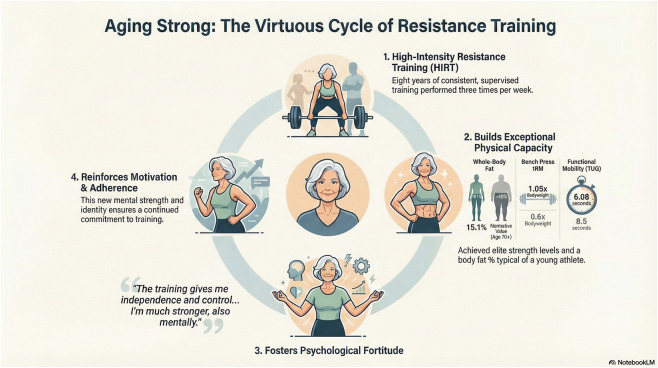
Conceptual virtuous-cycle of aging strong with long-term high-intensity resistance training (HIRT), illustrating the interplay between physical adaptations, psychological processes, and qualitative experiences observed in this case.

The trainer–trainee relationship was described as an intimate, accountability-providing connection *(“without a trainer, many people quit”)*, echoing evidence that professional supervision and social support promote exercise adherence in older adults (Rivera-Torres et al., 2019; Collado-Mateo et al., 2021). Training also served as a primary coping strategy, improving emotional balance, cognitive clarity, and resilience during stress and loss, consistent with literature linking exercise to better mood and psychological resilience in aging (Cunha et al., 2024; Churchill et al., 2022). Social effects extended beyond the individual, as family members adopted healthier behaviors in response to her example, highlighting how a highly engaged older exerciser can shape the surrounding social environment and support long-term adherence (Rivera-Torres et al., 2019; Collado-Mateo et al., 2021). Taken together, the results indicate a dynamic interaction among physiological, psychological, and social domains: progressive, long-term HIRT increases strength, power, and functional capacity; these gains enhance self-perception, emotional balance, and a positive sense of identity; and these psychological resources, supported by a strong trainer–trainee relationship and training-centered identity, reinforce adherence. From a broader geroscience perspective, this integrated profile resonates with the WHO concept of *vitality capacity*, which emphasizes multisystem resources that underlie intrinsic capacity and functional ability in later life (Bautmans et al., 2022). In this case, long-term HIRT appears to support high musculoskeletal function, mobility, and psychological resilience simultaneously, offering a person-level illustration of how resistance training may contribute to vitality capacity in older adulthood. The participant’s exceptional strength, power, body composition, and functional performance co-occurred with high general self-efficacy, high self-esteem, unwavering adherence, empowered body image, and a central role for training in emotional regulation and everyday life *(“It’s part of me … part of my life*.*”)*. This integrated profile supports a virtuous-cycle in which progressive, individualized HIRT builds functional and psychological reserve, allowing independence and effective coping with stress, while greater confidence and independence further strengthen motivation and adherence. Figure 3 summarizes this virtuous-cycle of aging strong with long-term HIRT, illustrating an upper-bound model of how resistance training can function not only as a health behavior but as a core component of identity and autonomy in later life.

### Clinical and practical implications

Although this is a single highly trained case, it offers several practical implications for clinicians, trainers, and program designers (Izquierdo et al., 2025; Fragala et al., 2019). When appropriately screened and supervised, older adults can tolerate and benefit from long-term HIRT at intensities and volumes higher than those often prescribed in routine care (Liu and Latham, 2009). Programs should therefore avoid assuming low load ceilings solely on the basis of chronological age and instead emphasize progressive overload, multi-joint movements, and careful technique coaching (Izquierdo et al., 2025; Fragala et al., 2019).

The findings also reinforce the value of individualized prescription and monitoring. The mismatch between group-based MVTs and the participant’s true 1RM suggests that velocity-based or percentage-based loading schemes derived from less-trained samples may under-challenge highly trained older adults. Practitioners using velocity-based methods should therefore consider individual L-V profiling rather than relying only on published thresholds, especially with long-term trainees (Alcazar et al., 2018; García-Ramos, 2023).

Psychological and relational factors also appear central to sustained HIRT in later life (McAuley et al., 2011). The participant’s high self-efficacy, self-talk, and strong trainer-trainee relationship supported adherence and willingness to train near failure. Practitioners can use these insights by teaching self-talk, fostering a task-focused climate, and building stable, supportive coaching relationships (McAuley et al., 2011).​

Finally, the case underscores the role of environmental and lifestyle support. Access to a well-equipped home gym, high daily physical activity, and stable body weight likely helped sustain training and translate gains into everyday function. Clinicians and community programs aiming to promote “aging strong” should address practical barriers and support feasible home-based or community-based resistance training (Brogno, 2025; Thiebaud et al., 2014).

### Limitations

This study has several important limitations. It reports on a single, highly trained 71-year-old woman selected as an upper-bound exemplar, so the findings are not statistically generalizable to the broader older-adult population. The participant’s socioeconomic resources, access to a private trainer and well-equipped home gym, and long history of exercise likely distinguish her from many older adults, limiting external validity. The dual trainer–researcher role may have influenced both training and qualitative reporting, despite efforts to mitigate this through reflexive notes and independent review of themes. The design is cross-sectional, with no pre-training baseline data, so causal inferences about HIRT effects must be drawn cautiously. In addition, some mechanistic interpretations (for example, regarding neuromuscular efficiency or V_1_RM characteristics) rely on inference from existing literature rather than direct measurement.

Despite these constraints, the case provides a detailed, mixed-methods account of an upper-bound adaptation pattern that can inform hypothesis generation, exploration of potential mechanisms, and the design of applied interventions to support resistance training and successful aging in older adults.​​

### Conclusion

This single participant demonstrates that a 71-year-old woman can achieve exceptional physical and psychological functioning after 8 years of supervised HIRT, with strength, power, body composition, functional performance, self-efficacy, and self-esteem all exceeding typical age-matched expectations. Although this represents an upper-bound phenotype rather than a realistic target for most older adults, it illustrates the magnitude of adaptation that may be possible when resistance training is progressive, individualized, and sustained over many years.​

The mixed-methods findings suggest that long-term HIRT can do more than preserve muscle and function; in selected older adults, it may also support psychological resilience, positive body image, and a strong sense of identity and independence. For clinicians, exercise professionals, and community or rehabilitation programs, this case highlights several modifiable levers that can be embedded into resistance-training programs for older adults: careful screening, high-quality supervision, progressive loading, deliberate use of self-regulation strategies (such as self-talk), and practical support for regular training (for example, access to appropriate equipment and stable routines). Taken together, these elements outline how individualized HIRT can be used as a practical tool to help selected older adults move toward their genetic physical and psychological potential and, in doing so, “aging strong” in everyday life, provided that appropriate screening and close supervision are in place.

## Data Availability

The raw data supporting the conclusions of this article will be made available by the authors, without undue reservation.
